# Regulatory Roles of miR-155-5p, miR-21-5p, miR-93-5p, and miR-140-5p in Breast Cancer Progression

**DOI:** 10.3390/cimb47050377

**Published:** 2025-05-20

**Authors:** Mai S. Degheidy, Amany A. Abou-Elalla, Mahmoud M. Kamel, Shaimaa Abdel-Ghany, Borros Arneth, Hussein Sabit

**Affiliations:** 1 Department of Medical Laboratory Technology, Faculty of Applied Health Science Technology, Misr University for Science and Technology, Giza 12566, Egypt; 2 Department of Clinical Pathology, National Cancer Institute, Cairo University, Cairo 11796, Egypt; 3 Department of Environmental Biotechnology, College of Biotechnology, Misr University for Science and Technology, Giza 12566, Egypt; 4 Institute of Laboratory Medicine and Pathobiochemistry, Molecular Diagnostics, Hospital of the Universities of Giessen and Marburg (UKGM), Philipps University Marburg, 35043 Marburg, Germany; 5 Institute of Laboratory Medicine and Pathobiochemistry, Molecular Diagnostics, Hospital of the Universities of Giessen and Marburg (UKGM), Justus Liebig University Giessen, 35392 Giessen, Germany; 6 Department of Medical Biotechnology, College of Biotechnology, Misr University for Science and Technology, Giza 12566, Egypt

**Keywords:** breast cancer, microRNAs, biomarkers, chemoresistance, paclitaxel

## Abstract

Breast cancer (BC) remains the leading cause of cancer-related morbidity and mortality worldwide, necessitating innovative approaches to improve diagnosis, prognosis, and treatment. This case-control study, aimed to evaluate the expression profiles of specific microRNAs (miRNAs)—miR-155-5p, miR-21-5p, miR-93-5p, and miR-140-5p—in 50 female BC patients treated with paclitaxel (PTX) compared to 50 healthy controls. miRNA expression was analyzed using qPCR. The study revealed significant up regulation of these miRNAs in BC patients, with miR-155-5p and miR-21-5p demonstrating the highest diagnostic accuracy (AUC = 0.890 and 0.863, respectively). These miRNAs are implicated in key oncogenic processes, including tumor growth, angiogenesis, metastasis, and chemoresistance, highlighting their potential as non-invasive biomarkers for BC diagnosis and prognosis. Additionally, the study identified significant differences in demographic and biochemical parameters between BC patients and controls, such as lower hemoglobin and RBC counts in patients, indicative of cancer-related anemia, and elevated AST levels. The findings underscore the importance of miRNAs in BC biology and their potential to guide personalized therapeutic strategies. Validation in larger cohorts is recommending and exploring miRNA-based interventions to improve patient outcomes and overcome chemoresistance in BC.

## 1. Introduction

Breast cancer (BC) has emerged as the most diagnosed cancer globally, surpassing lung cancer in incidence. In 2020, BC accounted for 2.3 million new cases, representing 11.7% of all cancer diagnoses, and was responsible for 685,000 deaths worldwide [1]. Among women, BC is the leading cause of cancer incidence in most countries and the primary cause of cancer mortality in 110 nations. In Egypt, BC is the most prevalent malignancy among women, with incidence rates increasing by over 23% in the past six years. Despite advances in treatment, survival rates in Egypt remain low, ranging from 28% to 68%, highlighting the need for improved diagnostic and therapeutic strategies [2,3].

BC is a heterogeneous disease originating from different cell types within the breast, including ductal and lobular cells. Its complexity is further compounded by molecular and physiological variations, which influence treatment outcomes. The disease is classified based on histological grading, tumor stage, and the expression of key biomarkers such as estrogen receptor (ER), progesterone receptor (PR), and human epidermal growth factor receptor 2 (HER2) [4,5,6]. Treatment strategies vary according to molecular subtypes, with endocrine therapy, chemotherapy, anti-HER2 therapy, and immunotherapy being the mainstay of management [7,8].

Chemotherapy, particularly paclitaxel, remains a cornerstone of BC treatment. Paclitaxel, a member of the taxane family, is widely used for its potent antitumor effects. However, the development of chemoresistance—either acquired or de novo—poses a significant challenge, leading to treatment failure and poor patient outcomes [9,10]. Resistance mechanisms include drug inactivation, target alteration, DNA damage repair, and epithelial-mesenchymal transition (EMT), underscoring the need for novel therapeutic targets.

Epigenetic modifications, including DNA methylation, histone modifications, and microRNA (miRNA) regulation, play a pivotal role in BC pathogenesis. These reversible changes influence gene expression without altering the DNA sequence, making them attractive targets for therapeutic intervention [11,12]. miRNAs have garnered significant attention due to their ability to regulate gene expression post-transcriptionally. These small non-coding RNAs modulate key cancer hallmarks, including cell proliferation, apoptosis, invasion, metastasis, and drug resistance [13,14,15].

miRNAs are detectable in various bodily fluids, including blood, plasma, and saliva, making them promising non-invasive biomarkers for early diagnosis, prognosis, and treatment monitoring [16,17]. MiR-155 is encoded within and processed from an exon of the MIR155 host gene (MIR155HG), a non-coding RNA transcribed from the B-cell integration cluster (BIC) locus situated on human chromosome 21. MiR-155 is known to play key roles in various physiological and pathological processes in the human body, influencing breast cancer carcinogenesis by regulating the expression of target genes, including oncogenes and tumor suppressors involved in this process [18].

An expanding database of evidence suggests that miR-155’s activity in breast cancer (BC) is linked to BRCA1, as the absence of functional BRCA1 has been shown to elevate miR-155 expression levels, and conversely, by direct targeting of the miR-155 promoter [19]. Evidence indicates that miR-155 can inhibit RhoA, FOXO3A, and SOCS1, hence facilitating epithelial-mesenchymal transition (EMT), cellular survival, proliferation, plasticity, and resistance to chemotherapy and radiotherapy in breast cancer [20].

miR-21 is extensively conserved and has been identified in 37 species. is situated on chromosome 17q23.2, within the 11th intron of the TMEM49 (transmembrane protein 49) gene [21], and has been associated with cell proliferation via several of its targets, including programmed cell death protein 4 (PDCD4), sprouty RTK signaling antagonist 2 (SPRY2), phosphatase and tensin homolog (PTEN), and reversion-inducing cysteine-rich protein with Kazal motifs (RECK) [22].

MiRNA-93 is encoded by a gene on chromosome 7q22.1. They are expressed in the nucleus transcribed with the host mini chromosome maintenance complex component 7 *(MCM7*) gene. It is a paralog of the miRNA-17–92 cluster, a member of the pro-oncogenic miRNA-106b-25 cluster. This cluster has been shown to regulate the expression of various target genes involved in important cellular processes such as cell proliferation, apoptosis and angiogenesis [23].

It has been revealed that STAT signaling pathway undergoes deregulation in a variety of disorders, particularly cancer. It seems that STAT3 is a target of miR-93 in breast cancer cells. LncRNA H19 inhibits the downregulation of miR-93 to enhance the expression of STAT3 signaling pathway leading to the increased proliferation and metastasis of breast cancer cells [21].

Research indicates that miRNA-93 is upregulated in various cancers, including breast cancer, lung cancer, colorectal cancer, prostate cancer, and pancreatic cancer. In these tumors, miRNA-93 functions as an oncogenic miRNA, facilitating tumor development and metastasis by modulating the expression of targeted genes associated with cell proliferation, angiogenesis, and invasion [24].

MiR-140 is one of the miRNAs that regulate the cell cycle and cell proliferation in a wild-type p53-dependent way. MiR-140 influences the cell cycle by reducing the S phase and promoting cell cycle arrest in the G0/G1 phase through the down-regulation of histone deacetylase 4 (HDAC4) [25].

Dysregulated miRNAs can act as oncogenes (oncomiRNAs) or tumor suppressors, influencing tumor growth, progression, and therapeutic response. For instance, miR-155 and miR-21 have been implicated in promoting chemoresistance, while miR-140 and miR-93 are associated with tumor suppression and drug sensitivity [26]. The ability of miRNAs to modulate drug resistance pathways highlights their potential as predictive biomarkers and therapeutic targets.

Despite significant advances in understanding the role of miRNAs in breast cancer (BC), previous studies have primarily focused on individual miRNAs and explored their roles in cell line experiments rarely on clinical samples. Additionally, limited research has systematically evaluated the diagnostic accuracy of miRNAs, particularly miR-155-5p, miR-21-5p, miR-93-5p, and miR-140-5p, in predicting paclitaxel resistance. So given the critical role of miRNAs in BC biology, studying their expression profiles and regulatory mechanisms is essential for advancing precision medicine. By identifying miRNA signatures associated with specific BC subtypes and treatment responses, researchers can develop targeted therapies to overcome chemoresistance and improve patient outcomes.

This study aims to explore the expression of key miRNAs—miR-155-5p, miR-21-5p, miR-93-5p, and miR-140-5p—in BC patients treated with paclitaxel, shedding light on their potential as diagnostic and prognostic biomarkers.

## 2. Subjects & Materials and Methods

### 2.1. Study Sample

This case-control study was conducted during the period from March 2023 to March 2024 at the College of Biotechnology, Misr University for Science and Technology. Blood samples were collected from 50 female breast cancer patients (age range 18–70) who attended to Baheya Foundation for Early Detection and Treatment of Breast Cancer (Giza, Egypt).

### 2.2. Inclusion and Exclusion Criteria

The study included neoadjuvant patients aged 18–70 years treated with paclitaxel (PTX) as a single agent administered weekly at a dose of 80 mg/m^2^ IV over three hours with no further chemotherapeutic drugs provided. Exclusion criteria comprised individuals with multiple tumors, liver disease, renal failure, peripheral neuropathy, vascular complications due to hypothyroidism, or autoimmune diseases. The control group consisted of 50 healthy participants were randomly selected from healthy women who visited the hospital for routine examinations. The inclusion criteria for control subjects were the absence of history of breast cancer or other malignancies as exclusion criteria, matched for age, BMI and gender with the patient group to ensure comparability.

### 2.3. Sample Size

The required sample size was calculated using G Power software version 3.17 for sample size calculation (Heinrich Heine Universität, Düsseldorf, Germany). In the patient group, the sample size of 50 subjects and 50 subjects in the control group was determined to provide 95% power for the *t*-test at the level of 5% significance. The effect size used for G Power analysis was 0.73.

### 2.4. Ethical Consideration

Informed consents were obtained from all participants, and the protocol was approved by the Baheya IRB: 202302200008 on 20 February 2023.

### 2.5. Data Collection

Data collection was conducted using a structured questionnaire comprising two parts. Part 1 focused on demographic information, including name, age, sex, residence, occupation, family history, smoking habits, and age of onset. Part 2 addressed medical history, capturing details such as the duration of breast cancer, current treatment, duration of disease, complications, side effects, tumor grade, tumor stage, and the onset of medication.

### 2.6. Equipment and Chemicals

The study utilized the Automated Chemistry ERBA XL-180 system (ERBA DIAGNOSTIC GmbH, Heidelberg, Germany) for laboratory analyses. Blood samples were collected using 5 mL vacuum tubes (HI VAC, Zhejiang, China). Urea, creatinine, calcium (total and ionized), alkaline phosphatase, uric acid, bilirubin (total and direct), albumin, Aspartate aminotransferase (AST), and Alanine aminotransferase (ALT) were analyzed using reagents from Erba Mannheim XL-180, Mannheim, Germany. The potassium kit was sourced from the Electrolyte Analyzer Genrui GE-300, Shenzhen China. PT kit was obtained from Spectrum, Cairo, Egypt. Chloroform and ethanol were provided by Piochem, Cairo, Egypt. For miRNA analysis, the miRCURY LNA RT Kit, MiRCURY LNA SYBR Green PCR Kit, miRNeasy Kit, QIAzol^®^ Lysis Reagent, and RNase-free water were all procured from QIAGEN, Hilden, Germany. MicroRNAs analyzed in the present study were hsa-miR-155-5p hsa-miR-21-5p hsa-miR-93-5p hsa-miR-140-5p and hsa-miR-103a-3p served as an endogenous control

### 2.7. Blood Sample Collection

Venous blood samples (5 mL) were collected from breast cancer patients and controls for complete blood count; prothrombin time; and partial thromboplastin time. The serum was separated for biochemical analysis; while EDTA-treated samples were stored at −80 °C for miRNA extraction

### 2.8. Hematological Tests

Hematological tests, including Complete Blood Count (CBC), were performed using the Sysmex analyzer (Kobe, Japan). Prothrombin Time (PT) was measured to evaluate coagulation pathways. PT was determined by adding thromboplastin to prewarmed plasma, then the time for clot formation was recorded automatically.

### 2.9. Biochemical Analyses

Biochemical tests, including the determination of creatinine, urea, uric acid, calcium, potassium, alkaline phosphatase, albumin, bilirubin (total and direct), alanine aminotransferase (ALT), and aspartate aminotransferase (AST), were performed automatically using the Erba Mannheim XL-180 analyzer (Mannheim, Germany). Electrolyte levels, specifically potassium, were measured using the Electrolyte Analyzer Genrui GE-300 (Shenzhen, China), ensuring accurate and automated assessments.

### 2.10. Pathological Data

Comprehensive pathological and diagnostic medical reports were obtained for each patient from the laboratory of Baheya Hospital (Cairo, Egypt). These reports included critical details such as patient age, tumor type, tumor grade, and the hormonal receptor status of estrogen and progesterone, providing valuable insights for the study.

### 2.11. Extraction of miRNA 

Total RNA, including miRNA, was extracted from EDTA blood samples of patients and controls using QIAzol^®^ Lysis Reagent (QIAGEN, Hilden, Germany). The miRNeasy Mini Kit utilizes phenol/guanidine-based lysis and silica-membrane purification to isolate RNA while removing DNA and proteins. The procedure involved multiple steps, including cell lysis, phase separation with chloroform, ethanol precipitation, and RNA purification through RNeasy Mini columns, resulting in high-quality RNA for further analysis.

### 2.12. Reverse Transcription and qPCR

The expression of miRNAs was analyzed through two steps: reverse transcription to synthesize complementary DNA (cDNA) using miRNA-specific stem-loop RT primers, followed by real-time PCR. RNA samples were diluted to 5 ng/µL using RNase-free water. A reaction mix was prepared on ice with the following components: 2 µL of 5 × miRCURY SYBR^®^ Green RT Reaction Buffer, 4.5 µL RNase-free water, 1 µL 10x miRCURY RT Enzyme Mix, 0.5 µL UniSp6 RNA spike-in (optional), and 2 µL template RNA. The total reaction volume was 10 µL. The mixture was incubated at 42 °C for 60 min to synthesize cDNA, followed by a 5-min incubation at 95 °C to inactivate reverse transcriptase, and then cooled to 4 °C.

The reverse transcription reactions were immediately used for real-time PCR, ensuring high specificity and sensitivity for miRNA detection. The process utilized universal thermal cycling conditions to amplify the target miRNAs from the synthesized cDNA. This approach offers accurate quantification of miRNA expressions, which is essential for understanding their role in disease mechanisms.

For miRNA expression analysis, the PCR reaction mix was prepared with the following components: 10 µL of 2x miRCURY SYBR^®^ Green Master Mix, 3 µL resuspended PCR primer mix, 2 µL of diluted cDNA template, and 5 µL RNase-free water. The total reaction volume for a single reaction was 20 µL.

The prepared reactions were mixed thoroughly, and 10 µL of the mix was dispensed into PCR tubes. The tubes were briefly centrifuged at room temperature before being placed in a real-time cycler. The PCR cycling conditions were set as follows: Initial heat activation: 95 °C for 2 min in maximal/fast mode. 2-step cycling: 40 cycles of denaturation at 95 °C for 10 s, followed by combined annealing/extension at 56 °C for 60 s with fluorescence data collection (SYBR^®^ Green). Melting curve analysis: 60–95 °C to assess specificity. Data analysis was conducted using real-time PCR instrument software (StepOne Plus v2.3) to calculate raw Ct values, which provide quantitative measures of miRNA expression. The relative expression of miRNAs was quantified using the comparative Ct (∆∆Ct) method, normalizing the target miRNA to a reference miRNA (hsa-miR-103a-3p) and comparing it to healthy controls. Fold change in expression was calculated as 2^−∆∆Ct^.

### 2.13. Statistical Analysis

Using SPSS (Statistical Package for Social Science) program for statistical analysis (version 26; Inc., Chicago, IL, USA). Descriptive data were expressed as mean μ and standard deviation (SD). Student *t*-test was used to compare the mean and SD of 2 sets of quantitative normally distributed data, Mann-Whitney U test to compare miRNAs. Significant differences were detected in the *p*-value. Confidence intervals (CI) were calculated to evaluate the relationship between the different cases—Spearman & Pearson correlation coefficient for non-parametric & parametric values. The ROC (Receiver Operating Characteristic) curve was used to detect the cutoff value with the highest sensitivity and specificity. The *p*-value was considered statistically significant when it was less than 0.05.

## 3. Results

### 3.1. Demographic Data for BC Cases and Control

**Figure 1:**
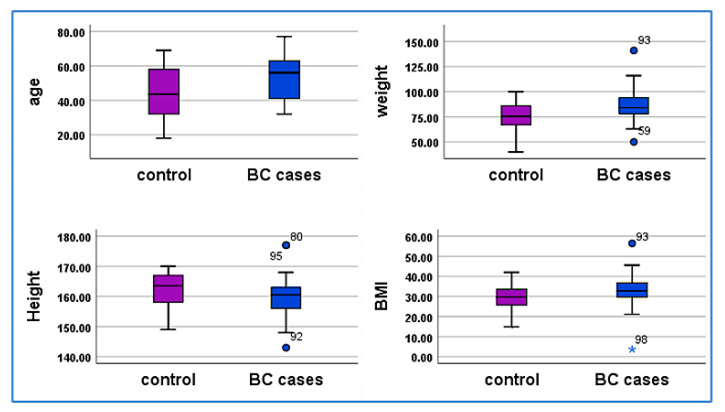
Box plots depict the Comparison of demographic variables (age, weight, height, and BMI) between 50 control and 50 breast cancer groups. Data points are presented as mean values, with purple box representing the control group and blue boxes representing the breast cancer group. The y-axis indicates fold expression levels. Significant differences are observed for age, weight, and BMI, while the difference in height is not statistically significant.

The demographic characteristics of the study groups—50 healthy women (control) and 50 women with breast cancer (case)—include age, weight, height, and BMI. The breast cancer group had a significantly higher mean age (53.12 ± 12.27 vs. 44.34 ± 14.84 years, *p* = 0.002), weight (86.64 ± 15.62 vs. 75.84 ± 14.07 kg, *p* < 0.001), and BMI (33.14 ± 7.64 vs. 29.02 ± 5.83, *p* = 0.003). Mean height was comparable between groups (159.94 ± 6.26 vs. 161.96 ± 5.63 cm, *p* = 0.093) and not statistically significant. Results are detailed in Table 1 and Figure 1.

### 3.2. Descriptive Data for Biochemical Analysis in BC Patients

The biochemical parameters in 50 women with breast cancer reveal significant variations. Hemoglobin (mean: 10.698 g/dL) and RBC count (mean: 3.9262 × 10^6^/µL) are below normal ranges, Hct (mean: 32.236%) while platelet count (mean: 306.120 × 10^3^/µL) shows notable variability. Potassium (3.970 mmol/L), ALP (61.020 U/L), calcium (4.354 mmol/L), and albumin (4.406 g/dL) levels are within acceptable ranges, while AST (26.880 U/L) and ALT (25.460 U/L) indicate mild liver enzyme elevation. Urea (26.740 mg/dL) and creatinine (0.823 mg/dL) are within normal limits, with slight variations observed across individuals, Total bilirubin (0.458 mg/dL) and coagulation parameters, including Pt (mean 12.637) INR (mean 1.007) (Table 2).

### 3.3. Descriptive Data for Biochemical Analysis in the Control Group

The control group of 50 healthy women shows biochemical parameters mostly within normal ranges. Hemoglobin (mean: 11.596 g/dL) and RBC count (mean: 4.4246 × 10^6^/µL) are in the normal range, with Hct averaging 32.568%. Platelet count (mean: 280.920 × 10^3^/µL) and WBC count (mean: 6.930 × 10^3^/µL) show expected variability. Potassium (mean: 3.830 mmol/L), ALP (59.060 U/L), calcium (4.2008 mmol/L), and albumin (4.168 g/dL) are within normal limits. ALT (21.520 U/L) and AST (21.840 U/L) levels suggest normal liver function, while urea (27.080 mg/dL) and creatinine (0.816 mg/dL) indicate normal renal function. Total bilirubin (mean: 0.821 mg/dL) and coagulation parameters, including Pt (12.620 s) and INR (1.006), remain within a healthy range. These data confirm the absence of pathological abnormalities in the control group (Table 3).

### 3.4. Biochemical Analysis in BC Patients and Control Group

**Figure 2:**
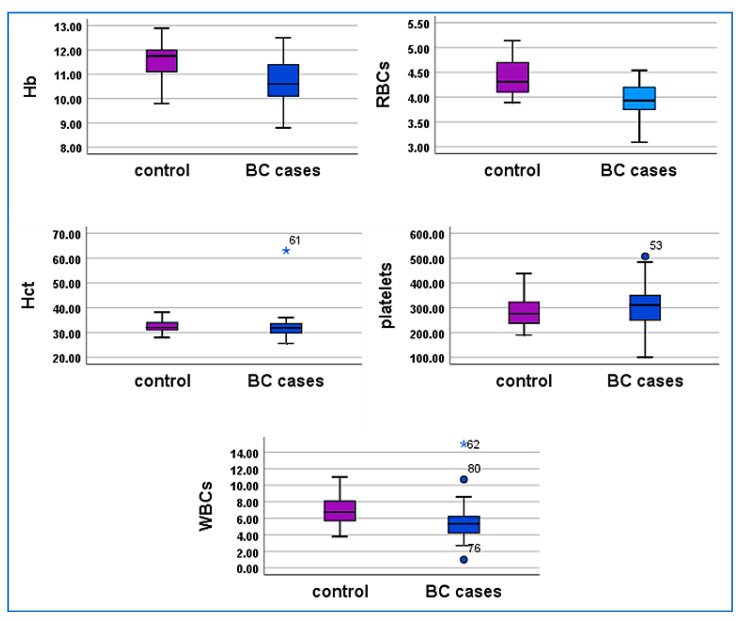
Comparison of Hematological Parameters Between 50 Breast Cancer Cases and 50 Controls: The box plot illustrates significant differences in hemoglobin, RBC, and WBC levels between the groups, while parameters such as PT and INR show no important variation. with purple box representing the control group and blue boxes representing the breast cancer group.

**Figure 3:**
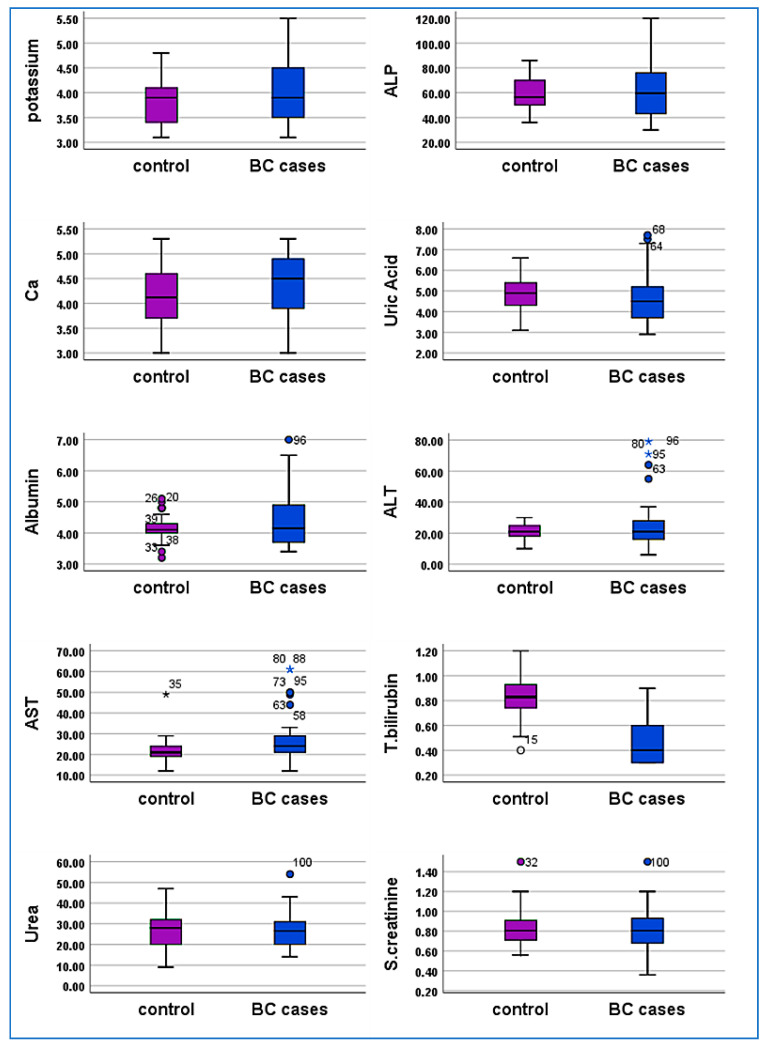
Box plot of Comparative Biochemical Parameters: The graph compares urea, serum creatinine, albumin, ALT, AST, T. Bilirubin, ALP and Ca levels between 50 breast cancer cases (blue) and 50 control group (purple). Statistically significant differences are noted in bilirubin and AST levels, while other parameters such as ALP, calcium, uric acid, potassium, and others show minimal variation.

The comparison of biochemical parameters between breast cancer cases and the control group reveals significant differences in several metrics. Hemoglobin, RBC count, and WBC count are significantly lower in cases, while AST levels are higher, all with *p* < 0.001. Total bilirubin is notably lower in cases, with *p* < 0.001, while other parameters like hematocrit, ALP, uric acid, calcium, and potassium show no significant differences between the groups (Table 4, Figure 2 and Figure 3).

### 3.5. Demographics and Basic Clinical Characteristics

Approximately 20% of patients reported a family history of breast cancer, while the majority (80%) had no such history. In terms of menopausal status, 64% of the patients were postmenopausal, and 36% were premenopausal. Tumor laterality analysis revealed that 56% of tumors were in the left breast, while 44% were in the right breast.

### 3.6. Tumor Characteristics

The type, grade, and mitosis score of the tumors highlighted the pathological and clinical variations in breast cancer. Invasive ductal carcinoma was the most common type, accounting for 92% of cases, with other types, such as invasive lobular carcinoma, representing only 2%. Histological analysis revealed that grade 2 tumors were the most prevalent (74%), followed by grade 3 (24%) and grade 1 (2%). Additionally, most tumors exhibited a mitosis score of 2 (88%) and a tubular formation score of 3 (72%), emphasizing their aggressive characteristics.

### 3.7. Receptor and Molecular Status

This category highlights the molecular characteristics of tumors, focusing on hormone receptor status and HER2 expression. Among the cases, 80% were ER-positive, 84% were PR-positive, and 82% were HER2-negative, with only 2% being HER2-positive and 16% classified as equivocal. Significant associations were identified between tumor grade and receptor statuses, showing a strong correlation with ER (*p* < 0.001) and a moderate correlation with PR (*p* < 0.001), underlining the molecular complexity and prognostic relevance of these markers.

### 3.8. Associations and Advanced Clinical Data

This category explores the interplay between tumor characteristics, stages, and treatment durations, providing valuable clinical insights. TNM staging revealed 46% of patients had no lymph node involvement (N0), while tumor sizes were predominantly at stages pT1 and pT2 (42% each). Treatment cycles ranged from 8 to 13 weeks, with 71% of patients undergoing 8-week cycles. Notably, tumor grade exhibited strong correlations with histological grade (*p* < 0.001) and nuclear grade (*p* < 0.001), alongside a significant association between tubular formation score and tumor grade (*p* = 0.001), emphasizing the interrelationship of these clinical parameters.

### 3.9. miRNAs Analysis

#### 3.9.1. miRNA 140-5p

The analysis of miR-140-5p reveals significant differences between the control group and breast cancer cases. In the control group, miR-140-5p levels exhibit a low mean and median, with minimal variance and a narrow range. In contrast, breast cancer cases display a markedly higher mean and median for miR-140-5p, indicating elevated levels in patients. The Mann-Whitney U test confirms this difference as statistically significant, with a *p*-value of less than 0.001. These findings highlight the potential of miR-140-5p as a biomarker, as its elevated levels are strongly associated with breast cancer cases compared to controls. The analysis of miR-140-5p reveals significant differences between the control and breast cancer cases. The control group exhibits a low mean (0.0969) and median (0.036) with minimal variance (0.016) and a narrow range (0.48).

**Figure 4:**
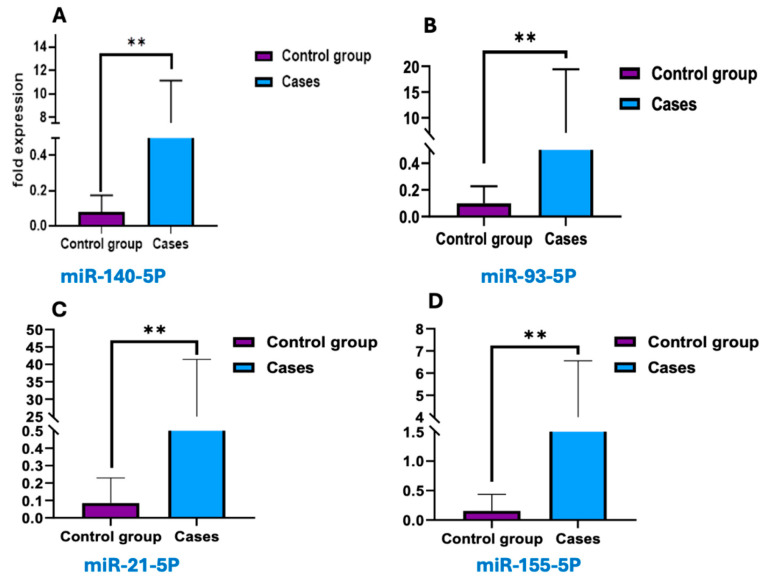
Comparative expression levels of miR-140-5p (**A**), miR-93-5p (**B**), miR-21-5p (**C**), and miR-155-5p (**D**) between 50 control and 50 breast cancer cases. Each graph shows significantly elevated miRNA expression levels in the breast cancer group compared to the control group. Panel (**A**) represents miR-140-5p, (**B**) miR-93-5p, (**C**) miR-21-5p, and (**D**) miR-155-5p, indicating their potential as biomarkers for breast cancer diagnosis. Statistical significance (*p* < 0.001) is observed across all panels, with error bars indicating standard deviation. ** Means that p value less than 0.001.

In contrast, the breast cancer cases demonstrate a substantially higher mean (3.2825) and median (0.882), with a large variance (61.706) and an extensive range (53.38). The standard deviation is notably higher in cases (4.692) compared to the controls (0.126). The Mann-Whitney U test value of 150 and a *p*-value of <0.001 confirm a statistically significant elevation of miR-140-5p in breast cancer cases compared to controls (Figure 4A).

#### 3.9.2. miRNA93-5p

Analysis of miRNA-93-5p levels reveals a significant disparity between the control group and breast cancer cases. In the control group, the mean and median miRNA-93-5p levels are relatively low at 0.0988 and 0.0404, respectively, with limited variation, as indicated by a standard deviation of 0.12934 and a variance of 0.017. The range of miRNA-93-5p values in the control group is narrow, spanning from 0.00 to 0.46. Conversely, breast cancer cases demonstrate a substantially higher mean and median miRNA-93-5p level, recorded at 5.0854 and 0.6186, respectively, accompanied by significant variation, reflected by a large standard deviation of 14.33970 and a variance of 205.627. The range of miRNA-93-5p values in breast cancer cases is notably broad, from 0.00 to 87.00. The Mann-Whitney U test result, with a *p*-value of <0.001, confirms a statistically significant difference in miRNA-93-5p levels between the two groups. These findings highlight the potential diagnostic relevance of miRNA-93-5p in distinguishing breast cancer cases from controls (Figure 4B).

#### 3.9.3. miRNA 21-5p

Analysis of miRNA-21-5p levels demonstrates significant differences between the control group and breast cancer cases. In the control group, miRNA-21-5p levels are consistently low, with a mean of 0.0535 and a median of 0.0191. The low standard deviation (0.102) and variance (0.011) indicate minimal variability within this group, and the range of values is narrow, spanning from 0.004 to 0.45. In contrast, the breast cancer cases show substantially elevated levels of miRNA-21-5p, with a mean of 1.2553 and a median of 1.2553. The high standard deviation (33.12863) and variance (1097.506) reflect considerable variability in miRNA-21-5p expression among patients. The range in breast cancer cases is extensive, from 0.0015 to 230.88, highlighting the broad variability in expression levels. The Mann-Whitney U test result, with a *p*-value of <0.001, confirms a statistically significant difference in miRNA-21-5p levels between the groups, suggesting its markedly higher expression in breast cancer cases (Figure 4C).

#### 3.9.4. miRNA-155-5p

Analysis of miR-155-5p reveals significant differences between the control group and breast cancer cases. In the control group, miR-155-5p levels are low, with a mean of 0.1247 and a median of 0.0173. The moderate variance (0.090) and standard deviation (0.299) suggest limited variability, with a narrow range from 0.0007 to 1.28. In contrast, breast cancer cases exhibit much higher miR-155-5p levels, with a mean of 1.8645 and a median of 0.3207. The large variance (22.018) and standard deviation (4.692) reflect significant variability, with an extensive range from 0.0004 to 29.66. The Mann-Whitney U test result (U = 166, *p* = 0.001) confirms a statistically significant elevation of miR-155-5p levels in breast cancer cases compared to the control group (Figure 4D).

**Figure 5:**
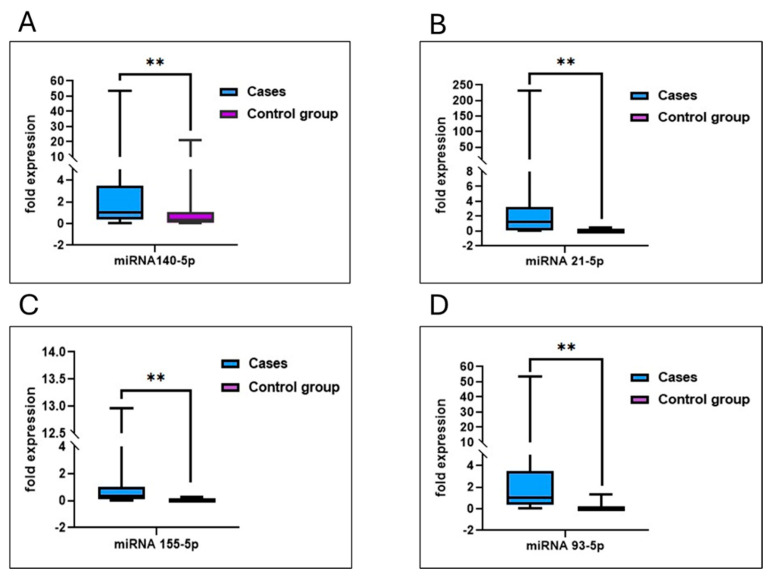
Differential expression levels of miRNAs between cases and control groups. Box plots depict the fold expression of miRNA-140-5p (**A**), miRNA-21-5p (**B**), miRNA(**C**), and miRNA-93-5p (**D**). The blue boxes represent 50 case groups, while the purple boxes represent the 50-control group. The y-axis indicates fold expression levels. Statistical differences between groups suggest distinct miRNA expression profiles associated with the studied condition, with notable upregulation or downregulation in the cases group compared to controls. Error bars represent variability within each group, highlighting significant variations between the cases and control groups. Data points represent the mean. ** Means that p value less than 0.001.

The expression profiles of miRNA-140-5p, miRNA-21-5p, miRNA-155-5p, and miRNA-93-5p were evaluated and compared between the case and control groups. As displayed in Figure 5, all four miRNAs were significantly elevated in the case group compared to controls (*p* < 0.01). Notably, miRNA-21-5p and miRNA-93-5p displayed the most significant increases. These findings show that the differential expression of these miRNAs may be Closely associated with the studied disorder and potentially contribute to its underlying biological mechanisms.

### 3.10. ROC Curve Analysis for miRNAs for BC Participants

#### 3.10.1. The AUC of miR-93-5p

**Figure 6:**
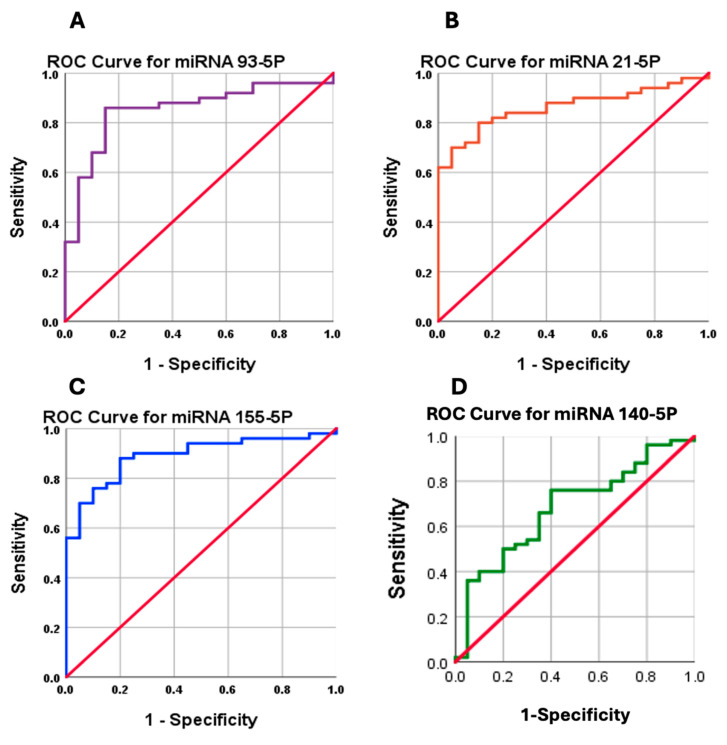
Receiver Operating Characteristic (ROC) curves for miRNAs 93-5P, 21-5P, 155-5P, and 140-5P, demonstrating their diagnostic performance in differentiating 50 breast cancer cases from 50 controls. Panel (**A**) (purple) shows miR-93-5P with an AUC of 0.853, Panel (**B**) (orange) displays miRNA 21-5P with an AUC of 0.863, Panel (**C**) (blue) illustrates miR-155-5P with an AUC of 0.890, and Panel (**D**) (green) represents miRNA 140-5P with an AUC of 0.680. Each ROC curve highlights the balance of sensitivity and specificity at various thresholds, with the diagonal red line representing random classification. The analysis indicates strong diagnostic potential for miR-93-5P, miR-21-5P, and miR-155-5P, while miR-140-5P shows moderate accuracy.

The analysis of miR-93-5p shows an Area Under the Curve (AUC) of 0.853, indicating excellent diagnostic accuracy. At a cutoff value of 0.05, the sensitivity is 86%, and the specificity is 85%. The *p*-value of <0.001 confirms that the AUC is statistically significant. The 95% Confidence Interval (CI) ranges from 0.757 to 0.949, further supporting the reliability of miR-93-5p as a diagnostic biomarker (Figure 6A).

#### 3.10.2. The AUC of miR-21-5p

The analysis of miR-21-5p reveals an Area Under the Curve (AUC) of 0.863, reflecting excellent diagnostic performance. At a cutoff value of 0.20, the sensitivity is 70%, and the specificity is 95%. The *p*-value of <0.001 confirms that the AUC is statistically significant. The 95% Confidence Interval (CI) ranges from 0.779 to 0.947, highlighting the robustness of miR-21-5p as a reliable biomarker for diagnostic purposes (Figure 6B).

#### 3.10.3. The AUC of miR-155-5p

The analysis of miR-155-5p demonstrates an Area Under the Curve (AUC) of 0.890, indicating strong diagnostic accuracy. At a cutoff value of 0.03, the sensitivity is 88,% and the specificity is 80%. The *p*-value of <0.001 confirms that the AUC is statistically significant. The 95% Confidence Interval (CI) ranges from 0.813 to 0.967, emphasizing miR-155-5p as a highly reliable biomarker for diagnostic evaluation (Figure 6C).

#### 3.10.4. The AUC of miR-140-5p

The analysis of miR-140-5p reveals an Area Under the Curve (AUC) of 0.680, indicating moderate diagnostic accuracy. At a cutoff value of 0.38, the sensitivity is 76,% and the specificity is 60%. The *p*-value of 0.019 confirms that the AUC is statistically significant. The 95% Confidence Interval (CI) ranges from 0.544 to 0.816, highlighting miR-140-5p as a potential diagnostic biomarker, though with less pronounced accuracy compared to other miRNAs analyzed (Figure 6D).

### 3.11. Spearman’s Correlation Among Different miRNAs

**Figure 7:**
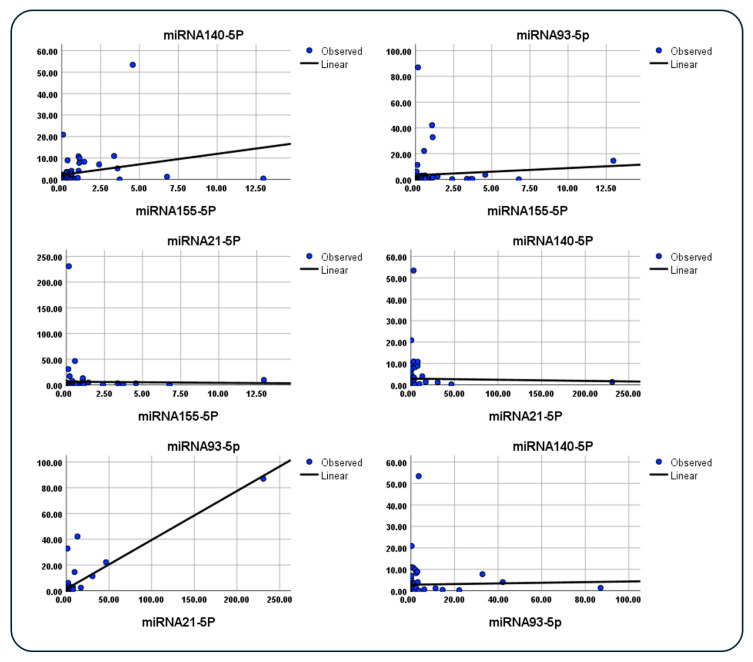
Correlation plots between various miRNAs, demonstrating the relationships observed in the study. **Top Left**: A moderate positive correlation is evident between miR-140-5p and miR-155-5p. **Top Right**: A strong positive correlation between miR-155-5p and miR-93-5p is highlighted. **Middle Left**: A weak positive correlation is observed between miR-155-5p and miR-21-5p. **Middle Right**: A strong positive correlation exists between miR-140-5p and miR-21-5p. **Bottom Left**: A weak positive correlation between miR-93-5p and miR-21-5p is shown. **Bottom Right**: A moderate positive correlation is displayed between miR-140-5p and miR-93-5p. Each plot represents observed values (blue dots) with their corresponding linear regression line (black line).

A moderate positive correlation is observed between miRNA 140-5p and miRNA 155-5p (r = 0.399, *p* = 0.004) as well as between miRNA 140-5p and miRNA 93-5p (r = 0.333, *p* = 0.018). Additionally, a strong positive correlation exists between miRNA 140-5p and miRNA 21-5p (r = 0.687, *p* < 0.001) and between miRNA 155-5p and miRNA 93-5p (r = 0.744, *p* < 0.001). Weaker positive correlations are noted between miRNA 155-5p and miRNA 21-5p (r = 0.298, *p* = 0.036) and between miRNA 93-5p and miRNA 21-5p (r = 0.209, *p* = 0.146) (Table 5, Figure 7).

### 3.12. Spearman’s Correlation Among the miRNAs and Biochemical Parameters

A strong positive correlation exists between miR-140-5p and hematocrit levels (r = 0.840, *p* < 0.001), as well as a moderate positive correlation with hemoglobin (r = 0.300, *p* = 0.034). However, miR-155-5p, 93-5p, and 21-5p do not show significant correlations with hematological parameters like RBCs, platelets, or WBCs. Similarly, no notable associations are found between the miRNAs and biochemical parameters such as albumin, ALT, AST, urea, and creatinine. These findings highlight miR-140-5p as the most relevant among the analyzed miRNAs in relation to hematological markers (Table 6).

### 3.13. Logistic Regression

**Figure 8:**
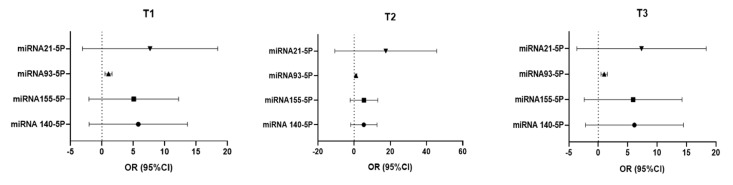
Forest plots illustrating the association between miRNA expression and tumor size categories (T1, T2, T3). Plots show Odds Ratios (OR) and 95% Confidence Intervals (CI) from logistic regression analyses for miR-21-5p, miR-93-5p, miR-155-5p, and miR-140-5p. The vertical line indicates an OR of 1.

The logistic regression analysis is performed to evaluate the association analysis between tumor size (T1, T2, T3) and expression levels of different miRNAs (miRNA140, miRNA155, miRNA93-5p, miRNA21-5p). The reference category is: ypT0. There was no statistically significant association between miRNA140, miRNA155, miRNA93-5p, or miRNA21-5p expression levels and tumor size (T1–T3) compared to tumor-free cases (T0). However, a non-significant trend toward increased tumor size with higher levels of miRNA140, miRNA155, and miRNA21-5p was observed (Table 7 and Figure 8).

## 4. Discussion

Breast cancer (BC) is a multifaceted and heterogeneous disease, ranking as the second most common cancer globally. According to recent estimates, there are approximately 2,261,419 new cases and 684,996 deaths annually, underscoring its significant public health burden [27]. The disease is influenced by a combination of genetic, lifestyle, and hormonal factors, with genetic predispositions such as BRCA1 and BRCA2 mutations playing a critical role in increasing susceptibility [28]. Despite substantial progress in understanding the genetic basis of BC, much of the research has historically focused on protein-coding genes, which constitute only about 2% of the human genome. This has left the vast non-coding regions, which are now emerging as key players in cancer biology, relatively understudied.

Globally, BC is the most prevalent malignancy among women, significantly impacting female health. Chemotherapy remains a cornerstone of BC treatment, but chemoresistance poses a major challenge, often leading to treatment failure or disease recurrence. Recent studies have highlighted the regulatory role of microRNAs (miRNAs) in chemosensitivity, positioning them as promising diagnostic and therapeutic targets [29]. miRNAs are increasingly recognized for their roles in BC progression, with growing evidence supporting their utility as biomarkers for diagnosis, prognosis, and treatment response prediction [16,30,31].

This study aimed to evaluate the expression profiles of specific miRNAs—miR-155-5p, miR-21-5p, miR-93-5p, and miR-140-5p—in BC patients treated with paclitaxel. The demographic analysis revealed significant differences between the control group (50 healthy women) and the BC group (50 patients) in terms of age. The mean age of the BC group was 53.12 years (±12.27), significantly higher than the control group’s mean age of 44.34 years (±14.84), suggesting that age is a critical risk factor for BC [32,33] according to the study highlights that the distribution of histopathologic and stage characteristics of presenting cancers vary significantly across the age spectrum, most evidently in the youngest age group and elderly group 80 year. The clinical pathological features and profiles by age described in this study are consistent with many previous reports in the literature While the absolute number of patients analyzed at both the young (<35 years, *n* = 377 (1.5%)) and elderly age groups (≥80 years, *n* = 2075 (8.5%) [34]. In our study, the age difference between cases and controls was minimal, with both groups being under 70 years old on average, and their clinical features were largely comparable. Additionally, the BC group had a significantly higher average weight (86.64 kg ± 15.62) and BMI (33.14 ± 7.64) compared to the control group (75.84 kg ± 14.07 and 29.02 ± 5.83, respectively), indicating a potential correlation between obesity and BC risk, consistent with previous studies [35,36,37]. The majority of breast cancer patients undergoing chemotherapy experience weight gain. Studies report that 89–96% of patients gain weight during treatment, with average increases ranging from 1.2 kg to over 4 kg. Some patients gain more than 5 kg, and a smaller proportion gain over 10 kg [38].

Higher BMI is generally linked to a reduced risk of premenopausal breast cancer, especially when BMI is measured in early adulthood. The strongest inverse association is seen for BMI at ages 18–24, with risk decreasing as BMI increases in this age group [39].

Hematological parameters also showed significant differences between the groups. The mean hemoglobin (Hb) level was lower in BC patients (10.69 ± 0.88 g/dL) compared to controls (11.59 ± 0.73 g/dL), likely due to cancer-related anemia. Similarly, red blood cell (RBC) counts were lower in BC patients (3.92 ± 0.35 × 10^6^/µL) than in controls (4.42 ± 0.35 × 10^6^/µL), reflecting the systemic impact of cancer. These findings are supported by [40], who identified anemia as a common complication in BC patients, often resulting from tumor-related bleeding, bone marrow invasion, or malnutrition. White blood cell (WBC) counts were also lower in BC patients (5.48 ± 2.154 × 10^3^/µL) compared to controls (6.93 ± 1.65 × 10^3^/µL), suggesting immune suppression, a phenomenon exacerbated by chemotherapy-induced leukopenia [41].

Biochemical analysis revealed significant differences in aspartate aminotransferase (AST) and total bilirubin levels between the groups. Elevated AST levels, indicative of tissue damage, were observed in BC patients, consistent with previous findings [42,43]. Bilirubin, an endogenous antioxidant, was lower in BC patients, aligning with studies by [44,45], which reported reduced bilirubin levels in BC patients compared to healthy controls.

Steroid hormone receptor status showed that 80% of tumors were estrogen receptor (ER)-positive and 84% were progesterone receptor (PR)-positive. Lymph node metastasis was present in 52% of cases, a critical factor for staging and prognosis [46,47].

This study also explored the role of miRNAs as biomarkers. miR-155-5p, miR-21-5p, miR-93-5p, and miR-140-5p were significantly upregulated in BC patients treated with paclitaxel. miR-155-5p, an oncogenic miRNA, showed the highest diagnostic accuracy (AUC = 0.890), consistent with its role in promoting tumor growth, angiogenesis, and chemoresistance [48,49].

It was also reported that miR-155-5p is a prime regulator in inducing epithelial-mesenchymal transition (EMT); the state at which the carcinoma cells lose epithelial characteristics and acquire cell mobility to achieve invasion and exhibited a significant upregulation in TNBC and can be used as an indicative marker for TNBC [50].

MiRNA-155 inhibits apoptosis in breast cancer cells. Its involvement in apoptosis may contribute to its carcinogenic potential by inhibiting caspase-3 function. The overexpression of miRNA-155 results in a significant reduction of tumor protein 53-induced nuclear protein 1, which can induce cell cycle arrest and apoptosis via caspase-3 activation.

The study by Li revealed a strong positive association between miR-155-5p expression levels and the paclitaxel resistance, as the expression levels of miR-155-5p were upregulated in resistant cells. So miR-155-5p was suggested to be a key regulator of paclitaxel resistance in tumor cells, as it increased cell viability and motility, and promoted resistance to paclitaxel-induced apoptosis [51].

Any studies have shown significant differential miRNA expression profiles in breast cancer tissues compared to those of non-cancerous tissue, Subsequent investigations demonstrated that the genetic alteration of miR-155-5p greatly influenced the cell response to paclitaxel by modulating TP53INP1 expression miR-155-5p was overexpressed and TP53INP1 was down-regulated in MCF-7/PR compared with MCF-7 cells. that miR-155-5p and the target gene TP53INP1 may have a role in influencing the sensitivity to paclitaxel therapy. further evaluated the possibility that miR-155-5p therapy could contribute paclitaxel-resistance through the suppression of TP53INP1 in paclitaxel-resistant breast cancer cells. To verify this possibility, TP53INP1 was identified as a direct target gene of miR-155-5p by bioinformatics analysis [51].

Similarly, miR-21-5p, implicated in drug resistance and tumor progression, was significantly elevated in BC patients (AUC = 0.863), aligning with findings by [52,53,54,55]. miR-93-5p and miR-140-5p also showed significant upregulation, with AUC values of 0.853 and 0.667, respectively, supporting their potential as diagnostic biomarkers [56,57,58,59].

41–44miR-21-5p expression was up-regulated in tissues and plasma-derived exosomes of BC patients and in the BC cells and exosomes of cell culture media [54]. The up-regulation of miRNA-21 results in the promotion of cancer growth, proliferation, invasion, angiogenesis, and metastasis via targeting of many genes involved in apoptosis and tumor suppression, including programmed cell death protein 4 (PDCD4), RAS p21 protein activator (RASA1), phosphatase tensin and homolog (PTEN), P53, B cell lymphoma 2 (Bcl2) and signal transducer and activator of transcription 3 (STAT3) [60].

Identifying breast cancer could significantly enhance illness prognosis, thereby necessitating the development of minimally invasive and readily detectable diagnostic biomarkers for breast cancer diagnosis. Numerous studies have explored the prospective potential of microRNA molecules as diagnostic biomarkers for breast cancer patients in both tissue and blood samples. Based on the data obtained, the results indicate that miR-21, miR-155, and miR-23a are significantly overexpressed in the plasma of breast cancer patients compared to healthy individuals. In comparison to miRNA expression in other sample types, the expression of miR-21, miR-155, and miR-23a in breast cancer tissue samples exhibits a trend analogous to that observed in patient plasma samples in this investigation. Analogous to the elevation of blood levels of miR-21 and miR-155 observed in Chinese ethnic groups due to their overexpression, these miRNA molecules contribute to the oncogenic progression of breast cancer [61].

This study has specific limitations, notably the limited sample size, which may affect the generalizability of the results. A restricted sample can diminish the statistical power of the research and may fail to encompass the complete range of variability found in larger groups. Consequently, prudence is necessary when analyzing these findings.

Subsequent research with bigger, more heterogeneous cohorts is crucial to authenticate these findings and confirm their relevance to wider populations. Additional research may be undertaken to assess the expression of alternative miRNAs as prospective diagnostic biomarkers for breast cancer. Identify biomarkers correlated with breast cancer using extensive patient samples. Additional research may be undertaken to assess the expression of circulating miRNA155-5p, miRNA21-5p, miRNA93-5p, and miRNA140-5p as potential diagnostic biomarkers for breast cancer within the Egyptian population, utilizing a substantial patient sample size.

This study underscores the importance of miRNAs as biomarkers for BC diagnosis, prognosis, and treatment response. The findings highlight the potential of miR-155-5p, miR-21-5p, miR-93-5p, and miR-140-5p as valuable tools for improving BC management and overcoming chemoresistance. Further research is needed to validate these findings and explore their clinical applications.

## 5. Conclusions

Breast cancer (BC) continues to be a major global health challenge, with significant implications for patient survival and quality of life. This study underscores the growing importance of microRNAs (miRNAs) in understanding and managing BC. Specifically, miR-155-5p, miR-21-5p, miR-93-5p, and miR-140-5p were found to be significantly elevated in BC patients undergoing paclitaxel treatment. These miRNAs play pivotal roles in tumor progression, metastasis, and resistance to chemotherapy, making them promising candidates for diagnostic and prognostic applications. For example, miR-155-5p and miR-21-5p exhibited strong diagnostic potential, with AUC values of 0.890 and 0.863, respectively, highlighting their utility as non-invasive biomarkers.

Additionally, their involvement in drug resistance mechanisms suggests they could serve as predictive markers for treatment response, paving the way for personalized therapeutic strategies. The integration of miRNA profiling into clinical practice holds great promise for improving early detection, risk assessment, and treatment outcomes in BC. Future studies should focus on validating these findings in larger, diverse populations and exploring therapeutic interventions targeting miRNA pathways to enhance patient care and overcome chemoresistance.

To completely ascertain the clinical value of miRNAs, it is imperative to validate these findings in bigger, more heterogeneous populations. Comprehensive validation studies are essential to verify the consistency and reproducibility of miRNA panels across diverse populations, hence ensuring their application in practical clinical applications.

## Figures and Tables

**Table 1:** Demographic data analysis for two studied groups.

	Control (N = 50)	Cases (N = 50)	*p*-Value
Age	44.34 ± 14.84	53.12 ± 12.27	0.002
weight	75.84 ± 14.07	86.64 ± 15.62	0.000
Height	161.96 ± 5.63	159.94 ± 6.26	0.093
BMI	29.02 ± 5.83	33.14 ± 7.64	0.003

**Table 2:** Descriptive Statistics of biochemical parameters for cases (N= 50).

Parameter	Range	Minimum	Maximum	Mean	Std. Deviation
Hb	3.70	8.80	12.50	10.6980	0.88444
RBCs	1.45	3.09	4.54	3.9262	0.35360
Hct	37.40	25.60	63.00	32.2360	5.12042
Platelets	407.00	100.00	507.00	306.1200	82.99610
WBCs	14.00	1.00	15.00	5.4836	2.15466
Potassium	2.40	3.10	5.50	3.9700	0.63479
ALP	90.00	30.00	120.00	61.0200	22.13824
Ca	2.30	3.00	5.30	4.3540	0.60650
Uric Acid	4.80	2.90	7.70	4.7260	1.26746
albumin	3.60	3.40	7.00	4.4060	0.87445
ALT	73.00	6.00	79.00	25.4600	14.62485
AST	49.00	12.00	61.00	26.8800	10.86962
T. bilirubin	0.60	0.30	0.90	0.4580	0.16174
Pt	0.90	12.50	13.40	12.6370	0.19764
INR	0.09	1.00	1.09	1.0074	0.02284
Urea	40.00	14.00	54.00	26.7400	8.11351
S. creatinine	1.14	0.36	1.50	0.8228	0.17829

**Table 3:** Descriptive Statistics of biochemical parameters for control (N = 50).

Parameter	Range	Minimum	Maximum	Mean	Std. Deviation
Hb	3.10	9.80	12.90	11.5960	0.73844
RBCs	1.25	3.89	5.14	4.4246	0.35885
Hct	10.20	28.00	38.20	32.5680	2.09542
Platelets	248.00	190.00	438.00	280.9200	55.10541
WBCs	7.20	3.80	11.00	6.9300	1.65088
Potassium	1.70	3.10	4.80	3.8300	0.48750
ALP	50.00	36.00	86.00	59.0600	13.20082
Ca	2.30	3.00	5.30	4.2008	0.61700
Uric Acid	3.50	3.10	6.60	4.9220	0.84498
albumin	1.90	3.20	5.10	4.1680	0.39250
ALT	20.00	10.00	30.00	21.5200	4.43658
AST	37.00	12.00	49.00	21.8400	5.28517
T. bilirubin	0.80	0.40	1.20	0.8212	0.15927
Pt	1.20	12.20	13.40	12.6200	0.18736
INR	0.09	1.00	1.09	1.0056	0.01950
Urea	38.00	9.00	47.00	27.0800	7.28947
S. creatinine	0.94	0.56	1.50	0.8162	0.16332

**Table 4:** Comparison of biochemical parameters between the control group and cases.

Parameter	Control (N = 50)	Cases (N = 50)	*p*-Value
Hb	11.59 ± 0.73	10.69 ± 0.88	<0.001
RBCs	4.42 ± 0.35	3.92 ± 0.35	<0.001
Hct	32.56 ± 2.09	32.23 ± 5.12	0.672
Platelets	280.92 ± 55.105	306.12 ± 82.99	0.077
WBCs	6.93 ± 1.65	5.48 ± 2.154	<0.001
Potassium	3.83 ± 0.487	3.97 ± 0.634	0.219
ALP	59.06 ± 13.20	61.02 ± 22.13	0.592
Ca	4.20 ± 0.617	4.35 ± 0.60	0.214
Uric Acid	4.92 ± 0.844	4.72 ± 1.267	0.365
albumin	4.16 ± 0.39	4.40 ± 0.874	0.082
ALT	21.52 ± 4.43	25.46 ± 14.62	0.071
AST	21.84 ± 5.28	26.88 ± 10.86	0.004
T. bilirubin	0.82 ± 0.15	0.45 ± 0.16	<0.001
Pt	12.62 ± 0.187	12.63 ± 0.197	0.660
INR	1.00 ± 0.019	1.00 ± 0.022	0.673
Urea	27.08 ± 7.28	26.74 ± 8.113	0.826
S. creatinine	0.81 ± 0.163	0.82 ± 0.178	0.847

**Table 5:** Correlations between the miRNAs studied among cases.

			miR-140-5p	miR-155-5p	miR-93-5p	miR-21-5P
Spearman’s rho	miR-140-5P	r	1.000	0.399	0.333	0.687
		p	--	0.004	0.018	0.000
	miR-155-5P	r	0.399	1.000	0.744	0.298
		p	0.004	--	0.000	0.036
	miR-93-5P	r	0.333	0.744	1.000	0.209
		p	0.018	0.000	--	0.146
	miR-21-5P	r	0.687	0.298	0.209	1.000
		p	0.000	0.036	0.146	--

Correlation is significant at the 0.01 level (2-tailed). Correlation is significant at the 0.05 level (2-tailed).

**Table 6:** Correlations among the miRNAs and biochemical parameters.

		miR-140-5P	miR-155-5p	miR-93-5p	miR-21-5P
Hb (g/dL)	r	0.300	−0.014	0.042	0.119
	p	0.034	0.924	0.774	0.412
RBCs (×10^6^/µL)	r	0.248	−0.072	−0.161	0.034
	p	0.082	0.618	0.264	0.817
Hct (%)	r	0.840	−0.039	0.016	0.229
	p	0.000	0.787	0.912	0.109
platelets (×10^3^/µL)	r	0.183	0.107	0.100	−0.077
	p	0.204	0.460	0.491	0.594
WBCs (×10^3^/µL)	r	0.129	0.113	0.093	0.009
	p	0.372	0.436	0.521	0.953
potassium (mmol/L)	r	−0.059	−0.145	−0.138	−0.128
	p	0.683	0.314	0.339	0.375
ALP (U/L)	r	−0.137	−0.162	−0.141	−0.008
	p	0.344	0.260	0.328	0.956
Ca (mmol/L)	r	0.188	0.027	−0.051	−0.211
	p	0.190	0.850	0.727	0.141
Uric. Acid (mg/dl)	r	−0.276	0.151	0.231	−0.105
	p	0.052	0.295	0.107	0.468
Albumin (g/dL)	r	−0.226	−0.018	−0.098	−0.200
	p	0.114	0.902	0.497	0.164
ALT (U/L)	r	0.017	−0.008	−0.024	−0.068
	p	0.907	0.954	0.869	0.639
AST (U/L)	r	−0.097	0.156	0.070	−0.088
	p	0.502	0.279	0.627	0.542
Bilirubin (mg/dL)	r	−0.011	−0.094	−0.113	−0.187
	p	0.940	0.517	0.433	0.193
PT (seconds)	r	0.073	−0.175	−0.099	−0.079
	p	0.617	0.224	0.492	0.586
INR	r	0.020	−0.095	−0.058	−0.091
	p	0.888	0.514	0.688	0.531
urea (mg/dL)	r	0.040	−0.259	−0.167	0.126
	p	0.784	0.069	0.247	0.382
creatinine (mg/dL)	r	−0.210	−0.005	0.090	−0.074
	p	0.144	0.970	0.533	0.611

Correlation is significant at the 0.01 level (2-tailed). Correlation is significant at the 0.05 level (2-tailed).

**Table 7:** The logistic regression between tumor size and expression levels of different miRNAs.

Tumor Size		B	*p*-Value	Odd Ratio	95% Confidence Interval for Exp(B)	
					Lower Bound	Upper Bound
T1	MiRNA140	0.818	0.392	2.267	0.348	14.772
	miRNA155	0.567	0.582	1.764	0.234	13.305
	MiRNA93.5p	−0.050	0.861	0.951	0.544	1.664
	MiRNA21.5P	1.085	0.601	2.961	0.050	173.561
T2	MiRNA140	0.747	0.435	2.110	0.324	13.759
	miRNA155	0.636	0.537	1.889	0.250	14.244
	MiRNA93.5p	−0.068	0.813	0.935	0.534	1.636
	MiRNA21.5P	0.893	0.668	2.442	0.042	143.534
T3	MiRNA140	0.867	0.368	2.380	0.361	15.712
	miRNA155	0.718	0.486	2.051	0.271	15.498
	MiRNA93.5p	−0.098	0.732	0.906	0.517	1.590
	MiRNA21.5P	0.711	0.767	2.037	0.019	223.302

The reference category is: T0.

## Data Availability

All data generated in this study and included in the main MS.
